# The impact of *NRG1* expressions and methylation on multifactorial Hirschsprung disease

**DOI:** 10.1186/s12887-022-03287-1

**Published:** 2022-04-20

**Authors:** Gunadi, Alvin Santoso Kalim, Marcellus, Nova Yuli Prasetyo Budi, Kristy Iskandar

**Affiliations:** 1 Pediatric Surgery Division, Department of Surgery/Genetics Working Group/Translational Research Unit, Faculty of Medicine, Public Health and Nursing, Universitas Gadjah Mada/Dr. Sardjito Hospital, 55281 Yogyakarta, Indonesia; 2 Department of Child Health/Genetics Working Group, Faculty of Medicine, Public Health and Nursing, Universitas Gadjah Mada/UGM Academic Hospital, Yogyakarta, 55291 Indonesia

**Keywords:** Epigenetic, Aberrant expression, Hirschsprung disease, Methylation pattern, *NRG1*

## Abstract

**Background:**

Hirschsprung disease (HSCR) is a complex genetic disorder characterized by the lack of ganglion cells in the intestines. A current study showed that the *NRG1* rare variant frequency in Indonesian patients with HSCR is only 0.9%. Here, we investigated the impact of *NRG1* expressions and methylation patterns on the pathogenesis of HSCR.

**Methods:**

This cross-sectional study determined *NRG1* type I (*HRGα, HRGβ1, HRGβ2, HRGβ3, HRGγ*, and *NDF43* isoforms), type II and type III expressions in both ganglionic and aganglionic colons of 20 patients with HSCR and 10 control colons by real-time polymerase chain reaction (qPCR). For methylation studies, we treated the extracted gDNA from 16 HSCR patients’ and 17 control colons with sodium bisulfate and analyzed the methylation pattern of *NRG1* exon 1 with methylation-specific PCR. The samples were collected and analyzed at our institution from December 2018 to December 2020.

**Results:**

*NRG1* types I, II and III expressions were upregulated (17.2-, 3.2-, and 7.2-fold, respectively) in the ganglionic colons compared with control colons (type I: 13.32 ± 1.65 *vs.* 17.42 ± 1.51, *p* < 0.01; type II: 13.73 ± 2.02 *vs.* 16.29 ± 2.19, *p* < 0.01; type III: 13.47 ± 3.01 *vs.* 16.32 ± 2.58, *p* = 0.03; respectively); while only type I (7.7-fold) and *HRGβ1/HRGβ2* (3.3-fold) isoforms were significantly upregulated in the aganglionic colons compared to the controls (type I: 14.47 ± 1.66 *vs.* 17.42 ± 1.51, *p* < 0.01; *HRGβ1/HRGβ2*: 13.62 ± 3.42 vs 14.75 ± 1.26, *p* = 0.01). Moreover, the frequency of partially methylated *NRG1* was higher in the ganglionic (81%) and aganglionic (75%) colons than in the controls (59%).

**Conclusions:**

Our study provides further insights into the aberrant *NRG1* expression in the colons of patients with HSCR, both ganglionic and aganglionic bowel, which might contribute to the development of HSCR, particularly in Indonesia. Furthermore, these aberrant *NRG1* expressions might be associated with its methylation pattern.

**Supplementary Information:**

The online version contains supplementary material available at 10.1186/s12887-022-03287-1.

## Background

Hirschsprung disease (HSCR) is a heterogeneous genetic disorder characterized by the absence of ganglion cells in the gastrointestinal tract, causing a functional obstruction. The most common classifications are short-segment HSCR, long-segment HSCR, and total colon aganglionosis [1, 2]. The incidence of HSCR in Indonesia is higher (3.1 cases per 10,000 live births) [3] than other populations (*vs.* 1.5, 2.1, and 2.8 cases per 10,000 live births in European, African, and Asian ancestry cases, respectively) [1, 2]. This difference might be caused by the genetic background of Indonesians, particularly the *RET* rs2435357 and rs2506030 risk alleles [4].

*NRG1* has been successfully established as a gene candidate for HSCR disease [5]. This genome-wide association study result can be confirmed with the *NRG1* variants in patients with HSCR from European and Chinese populations [6, 7], where those mutations downregulate the protein level of NRG1 and cause HSCR disease. However, a current study showed that *NRG1* rare variant frequency in Indonesian patients with HSCR is only 0.9% [8].

Moreover, expressions of genes involved in HSCR are influenced by epigenetic mechanisms, including methylation patterns [9–11]. *NRG1* hypermethylation has been associated with the downregulated *NRG1* expressions [12]. In contrast, one study showed that *NRG1* expression was not affected by the methylation status [13]. Moreover, while one study showed the aberrant *NRG1* expression in patients with HSCR compared to controls [13], Tang et al*.* [14] revealed no differences in overall *NRG1* expressions between patients with HSCR and controls. These conflicting results concerning the role of NRG1 expressions and methylation level on HSCR pathogenesis emphasize the need for confirmation in other populations, mainly Indonesian.

## Methods

### Patients

This cross-sectional study involved all patients diagnosed with HSCR with the age of < 18 years old, except those who had low quality of DNA or RNA. The samples of ganglionic and aganglionic colons of patients with HSCR were collected at definitive surgery, while the control colon samples were obtained at stoma closure from patients with anorectal malformation. The samples were collected and analyzed at our institution from December 2018 to December 2020.

The HSCR patients' and controls' parents signed a written informed consent form to be included in this study. The Medical and Health Research Ethics Committee of Faculty of Medicine, Public Health and Nursing, Universitas Gadjah Mada/Dr. Sardjito Hospital gave approval for this study (KE/FK/0111/EC/2020 and KE/FK/0880/2018).

#### Total RNA isolation and quantitative real-time polymerase chain reaction (qPCR)

We extracted the total RNA from colons of 20 patients with HSCR and 10 controls according to our previous study [15], followed by a qPCR to determine the *NRG1* expressions using all of the isoform’s primer sets from a previous study [6].

#### DNA isolation, bisulfite conversion, and methylation-specific PCR

The QIAamp DNA Mini Kit (QIAGEN, Valencia, CA) was used to extract the total DNA from 16 HSCR patients and 17 control colons. Subsequently, the total DNA was determined using a NanoDrop 2000 Spectrophotometer (Thermo Scientific, Wilmington, DE, USA). Only high-quality DNAs with the OD260/280 ratios of 1.8 to 2.0 were utilized for the subsequent experiment.

DNA genomic (500 ng) was treated with sodium bisulfite using EZ DNA Gold Methylation Kit (ZYMO, USA), then continued with PCR. *NRG1* exon 1 methylation was analyzed using the following primers as follows: methylated forward: 5’-GTTTTAGCGCGGTCGTTC-3’, methylated reverse: 5’-CGAACTCCGACTTCTTACCG-3’; unmethylated forward 5’-GTAGTGTGAGTGTTTTAGTGTGGTTG-3, unmethylated reverse: 5’CAAACTCCAACTTCTTACCA-3’. PCR products were then run on gel agarose 2% using fluorosafe. Positive methylation DNA controls used methylated samples with SssI methyltransferase (New England Biolabs, MA, USA) for the methylation-specific PCR (MS-PCR) [13].

### Statistics

The Livak (2^−ΔΔC^_T_) method was used to compare the *NRG1* expressions between both the ganglionic and aganglionic colons from patients with HSCR and control colons [15]. *NRG1* expression data were provided as mean values ± standard deviation (SD). The normality of the *NRG1* expression was determined by the Kolmogorov–Smirnov test. Independent t-tests were used to analyze the significant differences of *NRG1* expression between the groups. A *p*-value of < 0.05 was considered significant. The IBM Statistical Package for the Social Sciences (SPSS) version 23 (Chicago, USA) was used for all statistical analyses.

## Results

### Comparison of NRG1 expressions in HSCR and control colons

We determined *NRG1* type I (*HRGβ1, HRGβ2, HRGβ3, HRGγ*, and *NDF43* isoforms), type II and type III expressions in both ganglionic and aganglionic colons of 20 patients with HSCR and 10 control colons. qPCR showed that expressions of all *NRG1* isoforms, except *HRGα/NDF43,* were upregulated in ganglionic colons compared with control colons, including type I, type II, and type III (*p* < 0.01, < 0.01, 0.03, respectively) (Table 1).

**Table 1:** *NRG1* expressions in the ganglionic colon of patients with HSCR and control colons

*NRG1* isoform	HSCR (ΔC_T_ ± SD)	Control (ΔC_T_ ± SD)	ΔΔC_T_ (95% CI)	Fold change	*p*-value
Type I	13.32 ± 1.65	17.42 ± 1.51	-4.10 (-5.38-(-2.82))	17.2*	< 0.01*
Type II	13.73 ± 2.02	16.29 ± 2.19	-2.57 (-4.21-(-0.92))	3.2*	< 0.01*
Type III	13.47 ± 3.01	16.32 ± 2.58	-2.84 (-5.42-(-0.27))	7.2*	0.03*
*HRGβ1/ HRGβ2*	13.01 ± 1.09	14.75 ± 1.26	-1.74 (-3.04-(-0.44))	3.3*	0.01*
*HRGβ3*	3.50 ± 1.60	4.88 ± 1.47	-1.38 (-2.56-(-0.19))	2.6*	0.02*
*HRGα/NDF43*	13.78 ± 3.00	13.50 ± 2.72	0.28 (-2.17–2.72)	0.8	0.82
*NDF43*	16.80 ± 1.63	18.52 ± 1.15	-1.72 (-3.03-(-0.40))	3.3*	0.01*

^*^p-value < 0.05

Interestingly, in the aganglionic colons of patients with HSCR, only type I and *HRGβ1/ HRGβ2* isoforms were significantly upregulated in the HSCR samples compared to the controls (*p* < 0.01 and 0.01) (Table 2).

**Table 2:** *NRG1* expressions in the aganglionic colon of patients with HSCR and control colons

*NRG1* isoform	HSCR (ΔC_T_ ± SD)	Control (ΔC_T_ ± SD)	ΔΔC_T_ (95% CI)	Fold change	*p*-value
Type I	14.47 ± 1.66	17.42 ± 1.51	-2.95(-4.23-(-1.67))	7.7*	< 0.01
Type II	14.61 ± 2.43	16.29 ± 2.19	-1.69 (-3.56–0.18)	3.2	0.07
Type III	15.30 ± 2.80	16.32 ± 2.58	-1.02 (-3.47–1.44)	2.0	0.40
*HRGβ1/HRGβ2*	13.62 ± 3.42	14.75 ± 1.26	-1.13 (-3.75–1.48)	3.3*	0.01
*HRGβ3*	4.01 ± 0.94	4.88 ± 1.47	-0.87 (-1.77–0.03)	1.5	0.06
*HRGα/NDF43*	13.19 ± 3.97	13.50 ± 2.72	-0.31 (-3.29–2.68)	1.2	0.83
*NDF43*	16.53 ± 2.93	18.52 ± 1.14	-1.99 (-4.15–0.16)	3.9	0.07

^*^p-value < 0.05

### Comparison of NRG1 methylation level in patients with HSCR and control

**Fig. 1:**
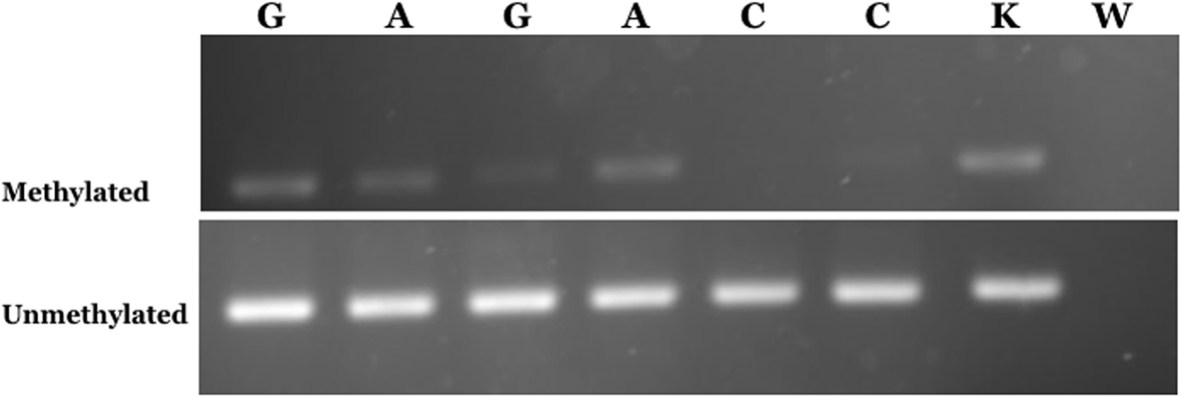
Representative electrophoresis result of *NRG1* methylation. G: ganglionic colon of HSCR patient; A: aganglionic colon of HSCR patient; C: control colon; K: positive control; W: negative control. Full-length gels are presented in Supplementary Figure 1. Positive control: positive methylation controls using DNA samples treated with SssI methyltransferase for the methylation-specific PCR (MS-PCR); negative control: without DNA

Next, we determined the *NRG1* methylation level in colons of 16 patients with HSCR and 17 controls. The frequency of partially methylated *NRG1* in the ganglionic (81%) and aganglionic (75%) colons were higher than the control (59%) colons, whereas none of the samples showed a full methylated pattern (Table 3 and Fig. 1).

**Table 3:** The methylation level of *NRG1* in colons of patients with HSCR and control

	**M (n, %)**	**M/U (n, %)**	**U (n, %)**
Ganglionic colon	0	13 (81)	3 (19)
Aganglionic colon	0	12 (75)	4 (25)
Control colon	0	10 (59)	7 (41)

*M* methylated, *M/U* partially methylated, *U* unmethylated

## Discussion

*NRG1* variants have been associated with the development of HSCR across populations [5–7]. However, the frequency of rare variants in our HSCR patients’ series is very low [8]. Therefore, we looked for other factors that might have a role in the HSCR pathogenesis, including *NRG1* expressions and methylation patterns.

Here, we are able to show the aberrant *NRG1* expressions in patients with HSCR, both in the ganglionic and aganglionic colons. Our study demonstrates significantly upregulated *NRG1* expressions in patients with HSCR compared with control colons, indicating that the aberrant *NRG1* expression might impact HSCR pathogenesis. This finding further confirmed a previous study [13]. However, our study has several novelties: 1) we tested the expressions of all isoforms of *NRG1*, including types I, II, and III (*vs.* only *NRG1* type I [13]); 2) we quantitatively compared the *NRG1* expressions between patients with HSCR and control colons (*vs.* only determined whether the *NRG1* was expressed in patients with HSCR and control colons [6]), and 3) in the Indonesian population (*vs.* Chinese population [6, 13]). Interestingly, although from the same Chinese population, two studies revealed different findings: one study had the aberrant *NRG1* expression in patients with HSCR [13], while another report [14] showed no differences in *NRG1* expressions between patients with HSCR and controls. These findings together with our results highlight the differences in the epigenetic profile in HSCR patients among population.

In addition, a previous study showed the downregulated *NRG1* expressions in breast cancer cell lines compared to normal ones [12]. In contrast, our results showed that *NRG1* expressions in Indonesian patients with HSCR are upregulated compared to controls. These differences might be due to 1) different diseases may have different impacts on *NRG1* expressions (developmental anomalies *vs.* cancer), 2) variations in genetic backgrounds between populations (Indonesia *vs.* Caucasian), and 3) different genetic resources (colon tissue *vs.* cell lines).

It has been shown that some gene expressions involved in the HSCR pathogenesis or enteric nervous system development are affected by the methylation pattern, including *RET, GFRA4, EDNRB,* and *SHH* [9]. Moreover, it has been hypothesized that *NRG1* expression was affected by its hypermethylation [13]. However, they failed to prove the hypothesis and suggested a further study on different ethnic groups. Here, we successfully showed that the partially methylated *NRG1* was higher in patients with HSCR than controls. Hypermethylation has been shown to suppress gene expressions [16]. Therefore, we suggest that the aberrant *NRG1* expressions in our patients might be due to the methylation status. The differences between our findings and a previous report [13] might be due to the differences in genetic characteristics among populations within Asian people [17].

Notably, limitations due to small sample size and single center study should be considered during interpretation of our findings. Moreover, it suggests a further multicenter studies with a larger sample size are necessary to identify other epigenetic factors that influence the *NRG1* expression in patients with HSCR.

## Conclusions

Our study provides further insights into the aberrant *NRG1* expression in the colons of patients with HSCR, both ganglionic and aganglionic bowel, which might contribute to the development of HSCR, particularly in Indonesia. Furthermore, these aberrant *NRG1* expressions might be associated with its methylation status.

## Supplementary Information

**Additional file 1.**

## Data Availability

All data generated or analyzed during this study are included in the submission. The raw data are available from the corresponding author on reasonable request.

## Abbreviations

*GAPDH*: *Glyceraldehyde-3-phosphate dehydrogenase*
HSCR: Hirschsprung disease
*NRG1*: *Neuregulin* *1*
qPCR: Quantitative real-time polymerase chain reaction
