# Supplementary figures and images for: The impact of NRG1 expressions and methylation on multifactorial Hirschsprung disease
Source: BMC Pediatr. 2022 Apr 20;22:216. doi: 10.1186/s12887-022-03287-1 (PMC9019992; doi:10.1186/s12887-022-03287-1)

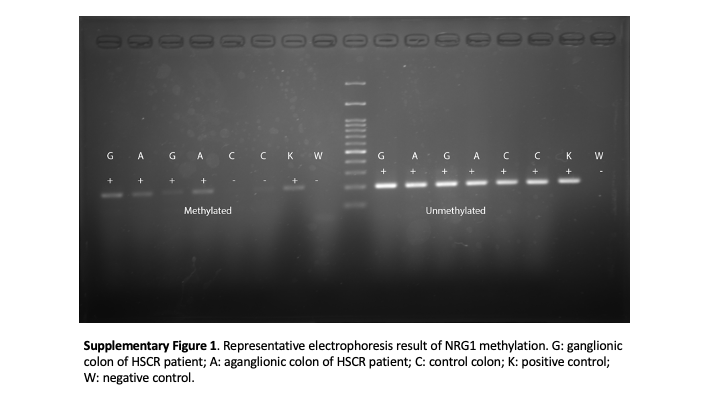

Supplement: Supplementary file 1 — Additional file 1. [file 12887_2022_3287_MOESM1_ESM.tiff]
